# Atlanta’s Potable Water

**Published:** 1883-08

**Authors:** 


### Editorial.

### ATLANTA'S POTABLE WATER.

Continued from Page 680 of July Number.

In the July number of the REGISTER, it was stated that we had examined, microscopically and chemically, the waters of several private wells, of the public wells, and the hydrant water of Atlanta; viz , the city hall park well, the well on Broad street, between Hunter and Alabama, the Markham House well, a well at 112 Decatur street and the popular well in Dr Boring's lot, on Forsyth street.

As a standard of purity, we also tested the water of Mr. McNaught's well, at the Southern end of Washington street, and that of another at the Western extremity of Fair street.

The water of the old city hall well is not only palatable, despite the irony taste from the pump, but is sparkling and perfectly pure from any organic contamination. It gave no indication to chemical tests of the least admixture or solution of organic matter. So that as regards purity, this well is as fair a standard as Mr. McNaught's, or the one at the end of Fair street.

The first of the central wells examined was the famous one in Dr. Boring's lot on Forsyth street. This water is sufficiently cool, sparkling and palatable, but it is not as pure as that which drops from the icicles on Diana's temple. It is rapidly becoming contaminated by the accumulating organic matter of this now crowded district of the city. There is not a particle of reason why it should escape the fate of others not far from it, that have been long abandoned because of their polution.

A microscopic view of this water reveals innumerable spores of vegetable organisms and bacteria. Under this test it is only less suspicious than the worst wells examined in tne city.

The results of a chemical test are of about the same nature. To nitrate of silver, after due acidulation to hold in solution any phosphate or carbonate of silver that might be formed, a very sensible cloudiness appeared. This indicates the presence of sodium chloride; and the question arises is this substance present here, in normal quantity, washed naturally from the soil and air by rain and present, more or less, in all such water; or has it been derived from the accumulations of surrounding organic matter? It may be from privies or cow sheds.

"Sewage, the polluting agent most to be dreaded in water, contains a large praportion of chlorine in the form of common salt, derived chiefly from the liquid excrements of animals." A satisfactory answer to the question would necessitate an exhaustive quantitative analysis, for which we have neither time nor material. We proposed only a proximate examination, and since neither the water of the city hall well, nor that of the McNaught well gives any such reaction with nitrate of silver, the result is just that far unfavorable to the theoretical purity of this well, and all others similarly situated in the city.

The Decatur street well, at 112, is exposed to the same sources of contamination; viz, sewage, surface accumulations of organic matter, and perhaps privies and stables. Its water, therefore, is more impure than that of the Boring well, in proportion of their greater intensity or closer proximity of these.

The microscope reveals the same spores of living organisms with a larger amount of other organic matters held in suspension. We saw in this water many of the large octahedron crystals of the chloride of sodium; a very sure evidence of sewage contamination. The chemical test gave a heavy precipitate of the chloride.

The next water examined was that of the Markham House well. This water is as largely used, perhaps, as that of the more public well on Broad street, South of Alabama street, and like the Boring well in comparison with itself, it is only not so impure as the well on Broad street. It lies, in this respect, about midway between them.

Beginning with the least contaminated, the wells rank in the following order: Boring's, Markham House, Decatur street and Broad street. The microscopic showing in this water is not less interesting than that of those previously examined, presenting the same living organisms and spores, though not so abundant as the Boring and Broad street waters. Chemically, the reaction gave evidence of a superabundance of chlorine derived from organic contamination.

The last of the central wells examined was that of Broad street, a little south of Alabama street. This well is the most central in its locality, and the most vitiated in the city. If the waters of the McNaught and old City Hall wells be marked zero in contamination, the waters of this would not be over estimated by 4 in the scale.

Yet this well is perhaps the most in use of all others in the city. The rattle of its pump handle never ceases. One of the principal soda-water founts in Atlanta gets its entire supply of water from this well. But then sparkling, foaming carbonic dioxide and the delicious syrups and flavorings effectually cover, if not a multitude of sins, myriads of vegetable spores and other organic dirt more dangerous to life and health.

It is an interesting, though not at all surprising fact, now, that beginning with the McNaught and other wells in the periphery of the city wheel, the drinkable water becomes more and more impure and unfit for use as the centre occupied by the Broad street well is approached.

The time has already come when the health department of the city would do a good thing to consider the question of an abundant supply of healthy, potable water for Atlanta. The problem is, where shall it be obtained? This question deserves another paper.

